# Communicating to Patients and Families About Post-Intensive Care Syndrome

**DOI:** 10.1016/j.chest.2025.01.024

**Published:** 2025-01-31

**Authors:** Mark L. Rolfsen, M. Elizabeth Wilcox, Matthew F. Mart, James C. Jackson, Carla M. Sevin, E. Wesley Ely

**Affiliations:** a Vanderbilt University Medical Center, Division of Allergy, Pulmonary and Critical Care Medicine, and The Critical Illness, Brain Dysfunction, and Survivorship (CIBS) Center, Nashville, TN; b Department of Critical Care Medicine, Faculty of Medicine & Dentistry, and the Neuroscience and Mental Health Institute, University of Alberta, Edmonton, Alberta, Canada; c Veteran Affairs Tennessee Valley Healthcare System Geriatric Research Education Clinical Center (GRECC), Nashville, TN

**Keywords:** family engagement, critical care, communication, mechanical ventilation, delirium, dementia, sedation, ICU Liberation, ABCDEF Bundle, respiratory failure, long-term outcomes, PICS, disability, functional impairment, brain injury

## Abstract

Millions of people around the world survive critical illness each year only to realize that they and their loved ones are grappling with a new “normal” after hospital discharge for which their medical team may not have adequately prepared them. Up to one-half of all ICU survivors suffer from new or worsening impairments in physical, cognitive, and psychological domains of health that are often not realized until they attempt to re-enter their previous lives. These devastating long-term sequelae of critical illness, collectively described as post-intensive care syndrome (PICS), can carry enormous consequences for an ICU survivor’s ability to care for their family, return to work, and regain their previous quality of life for months to years after their inciting illness. Despite mounting research on PICS and survivorship, a knowledge gap exists whereby ICU team members may not always be aware of PICS and may not counsel their patients on the challenges awaiting them after discharge. Understanding how best to communicate these challenges to patients and families is crucial in preparing for survivorship beyond the ICU. In this review, we summarize PICS and possible recovery trajectories of ICU survivors. We then discuss communication strategies, emphasizing the role of empathy. Finally, we provide a suggested framework to handle these crucial conversations. We aim to equip clinicians with the knowledge and framework to care for a patient who has survived critical illness but now faces the possibility of struggles inadequately addressed by our health care system.

## Cases to Frame Our “How I Do It”

### Case 1

A 41-year-old previously healthy woman is admitted to the ICU for ARDS. She is mechanically ventilated for 10 days, during which she receives deep sedation and neuromuscular blockade and remains immobilized. During and after mechanical ventilation, she suffers from hypoactive delirium. She is ultimately discharged to an inpatient rehabilitation facility, where she spends 2 weeks before returning home.

On returning home, she can dress and bathe her 2 young children but battles nightmares from her ICU stay and unrelenting intrusive memories that months later will be diagnosed as posttraumatic stress disorder (PTSD). Concentration is a challenge for her, and returning to her prior full-time job as a computer programmer is not possible. This places her household in financial peril. A primary care note reports normal abbreviated neurologic examination, and she is subsequently denied disability claims. Her general practitioner attempts to have her focus on being grateful for her survival and reassures her that all will be well if she just gives it time.

### Case 2

An 81-year-old man is admitted from home and diagnosed with septic shock from a biliary source. He requires mechanical ventilation and moderate doses of vasopressors. After 6 days he is extubated but remains delirious for 4 more days. He is discharged 2 weeks later to a subacute rehabilitation facility.

Although he previously enjoyed long walks with his dogs, he is now unable to walk 10 feet without a walker. He cannot remember his grandchildren’s names or do his daily crossword puzzle despite being “sharp as a tack” before his critical illness. The family is surprised at how challenging his life has become after critical illness, and many of them feel overwhelmed with new caregiver responsibilities. When they ask his primary care physician for an explanation of his problems in survivorship, they are told he may be getting Alzheimer disease. The patient’s daughter, who did not leave the ICU for most of his admission, experiences symptoms of depression with her increased caregiver responsibilities, which impedes her own life and functioning.

## Introduction

The field of critical care rapidly expanded starting in the 1950s, with advancing life support that reduced mortality for previously fatal organ dysfunction^1^; however, research into the long-term consequences of surviving these extremes of physiology did not begin in earnest until the 1990s.^2^^,^^3^ Since then, a large body of evidence has documented the impairments, disabilities, symptom burden, and health care costs associated with ICU survivorship, which can last for years.^4^^,^^5^ In 2012, the term post*-*intensive care syndrome (PICS) was first described to facilitate communication and research into impairments following critical illness—originally categorized into physical, cognitive, and mental health-related domains.^6^ Despite a quarter century of improvement in our understanding of the epidemiology of PICS, there is still poor awareness of the problem and infrequent communication between the medical team and patients.^7^^,^^8^

### Incidence

Although the incidence of PICS varies based on patient population, data from cohort studies suggest that it is common, occurring in up to 1 of every 2 ICU patients who survive to discharge. An analysis of 406 ICU survivors (median age, 61 years) found that only 44% were considered “PICS-free” at 12 months. At 3 months, 38% had cognitive impairment, 26% had physical disabilities with new limitations in activities of daily living, and 33% experienced symptoms of depression.^9^ A questionnaire-based study analyzed by ICU admission type showed the incidence of PICS at 1 year to be 43% for elective surgery requiring ICU admission, 58% for medical ICU admission, and 64% for urgent surgical ICU admission.^10^ Additionally, the term PICS-Family describes the wide-ranging and often underrecognized effects that an ICU admission can have on a patient’s caregivers and family. These include mental health problems such as anxiety, depression, and PTSD at rates similar to or exceeding those of the ICU survivor.^11^^,^^12^

### Recovery Trajectories and Risk Factors

Understanding a patient’s clinical and functional status before the ICU, including baseline frailty, disabilities, comorbidities, and age, is paramount in understanding the most probable trajectory of recovery.^13^^,^^14^ One study of 109 patients with ARDS with a median age of 44 years and few preexisting comorbidities found an 85% survival rate out to 2 years among those who survived to hospital discharge, with most returning to work and essentially all returning to independent living.^15^ In contrast, a cohort of 303 medical-surgical adult patients (median age of 62 years), who predominantly lived independently at home without preexisting physical or cognitive impairments, found that 43% had died within 6 months, and of the survivors less than one-third returned to their reported baseline functional status.^16^ At the upper end of age and frailty, Ferrante and colleagues^17^ described a large prospective community cohort assessed longitudinally for over a decade. Within this cohort, 291 adult patients (median age, 84 years) were admitted to an ICU and were categorized into 3 pre-ICU functional trajectories: minimal disability, mild to moderate disability, and severe disability. Both mortality and post-ICU functional status were worse with increasing pre-ICU level of disability. Overall, more than one-half experienced functional decline or early death. Finally, an observational study of over 1,500 ICU survivors emphasized this point further; among baseline characteristics, the strongest predictors for death within 1 year of ICU discharge were age, comorbidities, and loss of autonomy (ie, complete activities of daily living disability) at time of ICU admission.^18^

**Figure 1:**
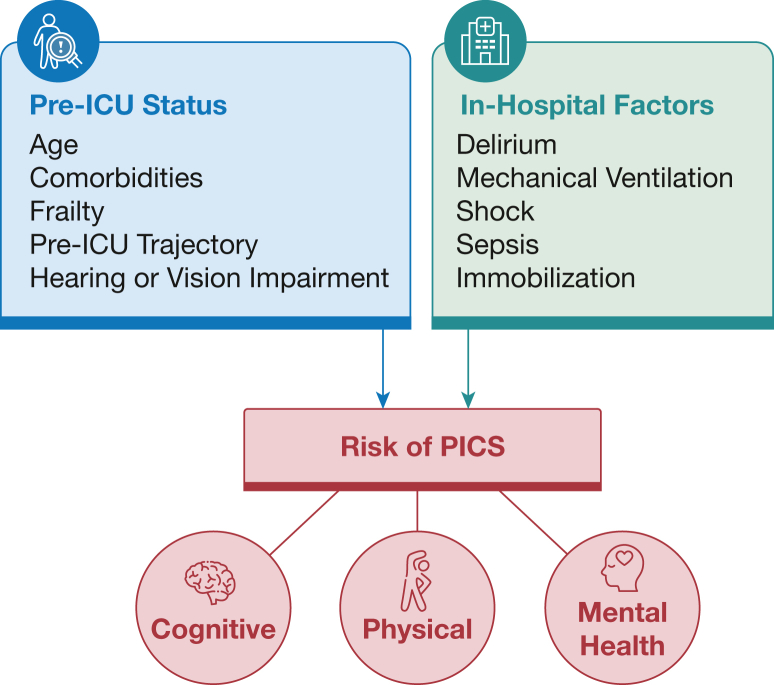
Major risk factors associated with development of PICS. PICS = post-intensive care syndrome.

Severity of illness and complications during an ICU stay also play a major role in long-term trajectories. Delirium duration is 1 of the strongest independent predictors of cognitive impairment and disability after discharge,^19^^,^^20^ emphasizing the need to prevent, monitor, and treat delirium to improve long-term outcomes. Length of mechanical ventilation, shock, and ICU length of stay (LOS) are also risk factors for decline in post-ICU functional status (Fig 1).^17^^,^^21^

Despite these known risk factors, few prediction models have been developed and validated. A recent systematic review found only 3 PICS-related prediction models.^22^ Since then, a model to predict the occurrence of PICS was developed and validated in a broad ICU cohort.^23^^,^^24^ Given providers’ low accuracy in predicting functional outcomes after the ICU,^8^ continued efforts at developing and implementing these models are necessary to help us advance our prognostication and communication to patients and families.

### Communication and Empathy in the ICU

Communication is a cornerstone of providing high-quality medical care, yet it has been recognized in both inpatient and outpatient medicine that physicians do not routinely discuss the long-term implications of serious illnesses.^25^ For example, more than one-half of patients with metastatic lung or colorectal cancer were unaware that chemotherapy would be unlikely to cure their disease.^26^ Although discussion of functional outcomes is often part of meetings between ICU providers and families,^27^ discordance in prognostic understanding is rife.^28^^,^^29^ Highlighting this fact, Cox and colleagues^30^ found that among 126 recipients of prolonged mechanical ventilation followed over 1 year, 83% of surrogates expected a good quality of life, whereas only 4% of physicians expected the same.^30^ Barriers to improving these pivotal conversations have been identified to include unrealistic surrogate optimism about recovery expectations, a lack of contact between intensivists and patients after discharge, and minimal confidence from intensivists in applying existing outcomes research to individual patients.^31^

Although evidence regarding best medical communication strategy is mixed,^32^ guidelines agree shared decision-making should start with information exchange and take into account the patient’s values and preferences.^33^ These values and preferences must be individualized, but qualitative data have suggested key themes that focus on avoiding functional impairments. In questionnaires of over 200 seriously ill outpatients, fewer participants reported they would choose treatments expected to result in physical or cognitive impairment than treatments that could result in death.^34^ Auriemma and colleagues^35^ likewise found in interviews with ICU survivors or surrogates that survival alone was inadequate as a goal. One-half focused on also maintaining physical or cognitive function as a meaningful outcome, with severe disability in these categories being considered a fate worse than death.^35^ These studies eloquently highlight what might be intuitive to many of us, that patients are humans who want to feel they have “a life worth living.”

**Figure 2:**
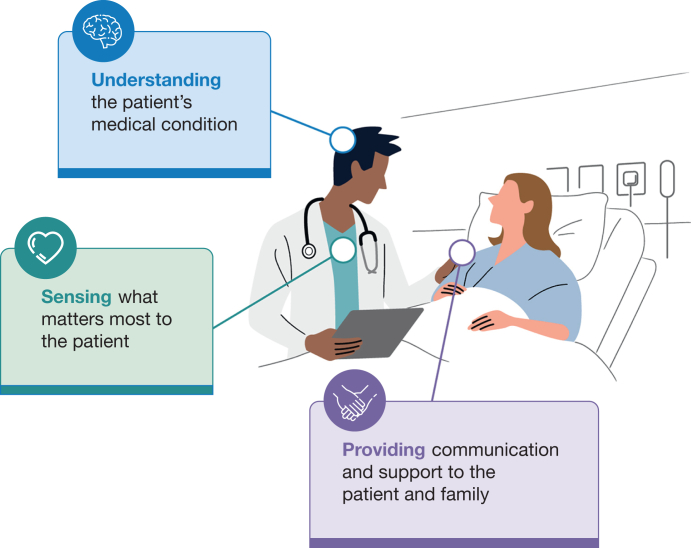
Components of empathy in ICU communication.

The topic of communicating recovery from critical illness would be incomplete without discussion about the importance of provider empathy. Sympathy is when we feel “for” someone; empathy is when we feel “with” someone. It can have benefits of patient satisfaction, improved communication with and acknowledgment of the individual patient, but also a degree of risk from “compassion fatigue.”^36^ Empathy in the ICU is a multifaceted concept and should include components of understanding a patient’s condition, sensing what matters most to them, and providing both support and communication (Fig 2).^36^ The first section of this article offers readers the understanding that patients’ recovery outcomes are influenced by their pre-ICU status and known risk factors. We then must genuinely inquire why the recovery matters to the patient. Is she an avid gardener, in which case meaningful recovery must include getting her hands in soil again? Is he a grandfather who finds joy in spending time with his grandchildren? What degree of recovery will be required to engage with them at a level he judges as meaningful? A willingness to have conversations about a meaningful life outside of the ICU provides a common goal for discussions on recovery. The third component, providing support and communication, is discussed next.

## How I Do It

**Figure 3:**
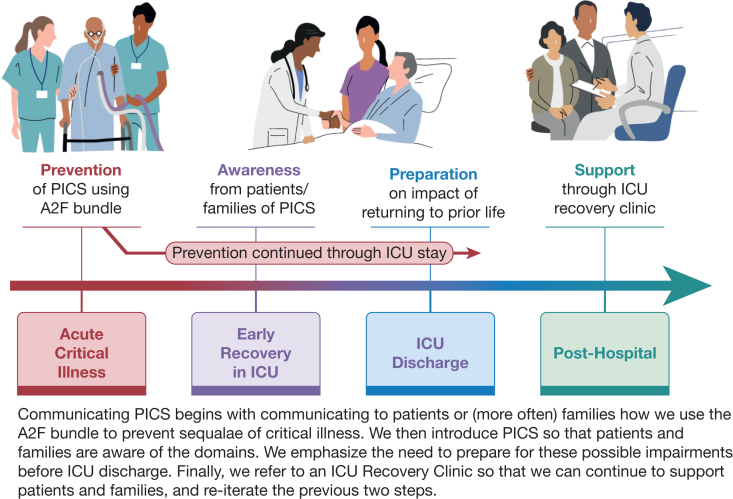
Timeline of communication PICS to patients and families. A2F bundle = ABCDEF bundle; PICS = post-intensive care syndrome.

As represented in our case examples, many patients receive inadequate preparation and discussion about common sequelae of critical illness. We pride ourselves on “saving lives,” yet too often we stumble at the finish line by leaving patients to discover for themselves the lingering effects of critical illness. Here we introduce a framework of how to improve this crucial component of ICU care, using an empathy-focused mindset incorporating prevention*,* awareness, preparation, and support (Fig 3).

**Figure 4:**
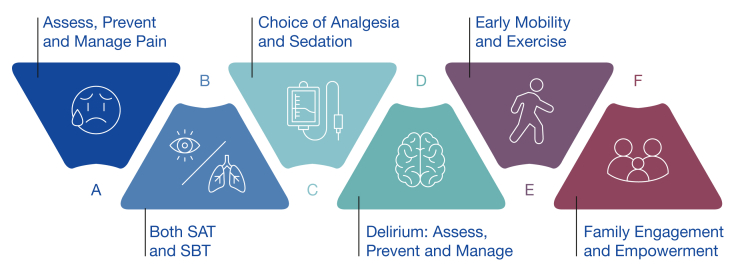
A2F bundle framework. A2F bundle = ABCDEF bundle; SAT = spontaneous awakening trial; SBT = spontaneous breathing trial.

The initial phase of a patient’s ICU course often includes critical stabilization and life-sustaining therapies. At that time, survival is often uncertain. Nevertheless, families are usually hopeful and want to know what steps we can take to improve the likelihood of survival and reduce the risk of long-term impairments. The ABCDEF bundle (aka, A2F bundle; Fig 4) is an evidence-based set of 6 steps that aim toward an “awake and walking” ICU culture.^37^ The A2F bundle is a way to bring a humanistic approach to ICU care with the backing of rigorous scientific evidence.^37^, ^38^, ^39^ Higher compliance with the A2F bundle’s 6 steps has been shown to reduce delirium, ICU LOS, mortality, and ICU readmissions in a dose-dependent manner.^38^^,^^39^ By decreasing delirium and ICU LOS, 2 variables mentioned as risk factors for PICS and worse functional trajectory following critical illness, we see this as a key component to decreasing the burden of PICS. Communicating the A2F bundle to families demonstrates that we are diligently providing holistic care and allows us to encourage their bedside participation. Higher family engagement during the COVID-19 pandemic decreased risk of delirium,^40^ and families should be aware of both the measurable and unmeasurable benefits of being at bedside when able.

For those patients in the ICU who survive their initial critical illness and have increasing likelihood of hospital survival, we begin to counsel patients and families on the recovery that lay ahead. Here, plain language and awareness of both health literacy and cultural differences is key in effective communication.^41^ We share that many ICU survivors face impairments or challenges after the hospital. We personalize these conversations to show empathy for the individual and an eye toward survivorship:

*I’m so glad that your father has improved from his critical illness. We’re hopeful that he’ll continue progressing towards leaving the ICU, but it’s important to be aware he might not immediately be capable of playing bridge or pickleball again because of the concentration and mobility problems many patients face after the ICU. We’ve been diligent about keeping him awake and walking daily to help him keep his muscles and brain as strong as possible so he’ll have less of these challenges*.

We include similar language in any major update to the family. As ICU discharge draws closer, we introduce more details while taking care to avoid jargon or overburdening the family. We review the various domains of PICS and emphasize that, although severity varies, these problems can occur in up to one-half of ICU survivors so that we normalize any impairments that may occur. We help prepare families by using the risk factors above to give qualitative assessments of possible recovery trajectories and take into consideration the patient’s initial characteristics—age, frailty, recent functional trajectory, and comorbidities—and in-hospital risk factors of delirium, sepsis, shock, mechanical ventilation and ICU LOS (Fig 1). We also emphasize that not every patient experiences PICS,^9^ and PICS-related symptoms can improve over months and years, admitting to the uncertainty inherent to predicting how any individual may recover.^42^^,^^43^ PICS affects more than patients, and we share with caregivers that they too may experience mental health difficulties and financial strain in primary caregiver roles.^12^ Before discharge, we discuss with patients and families the importance of post-hospital follow-up to address PICS and other challenges during the recovery period. We give anticipatory guidance to ease the transition of leaving the hospital and to help align expectations for what might be a new quality of life.^44^

Despite even the best attempts at these practices, patients and families often still leave the hospital with questions on medications, insurance, referrals, and more. ICU recovery clinics are multidisciplinary care teams that can emphasize all the prior points in the outpatient setting, where patients have had time to begin recovery. These clinics improve an ICU survivor’s reported quality of life and potentially decrease re-admissions by addressing the aforementioned problems, as well as providing access to peer support and palliative care.^45^, ^46^, ^47^, ^48^, ^49^ The multidisciplinary approach includes a pharmacist, psychologist, case manager, and ICU physician. Medications are reviewed and changes usually required, screening tools for PICS are performed, and referrals are placed when indicated. When a recovery clinic is not available, primary care providers must overcome barriers to caring for the complexities of a post-ICU patient^50^; we find that the awareness and preparation communicated to patients and families in the ICU can help but does not lessen the need for further research in improving recovery in the post-ICU period.

## Applying Our Framework

### Case 1

(A young, previously healthy woman who survived ARDS but suffers PTSD and cognitive impairment): *Ms*. *Smith, I can only imagine what you’ve been through these last 2 weeks*. *You were on the ventilator to* *support* *failing lungs*. *I’m so glad you’re getting better, but it’s important to remember that there is still a long road to full recovery ahead*. *Often it takes the body and brain longer to get well than it takes them to get sick*. *However, most people who come into the ICU at your young age, and in such previous good state of health, will return to close to their baseline or even back to baseline*. *Remember, though, that a large fraction will have impairments over the first several months, and some of these could even last longer*. *For you, that might mean difficulty immediately returning to your job as a computer programmer or remembering the details needed to manage your household with 2 kids*. *As an ICU survivor, you may develop mental health problems from the suffering you went through*. *We did our best to lower your risk of developing these problems by minimizing sedation and keeping you mobile*. *This is a lot to take in after what you’ve been through, and our ICU recovery clinic would be happy to see you after discharge*. *They can screen for these problems and provide available resources*. *You’re not alone in your recovery.*

### Case 2

(Older man with biliary septic shock who developed an accelerated dementia and mobility problems [Discussing with family because patient is still delirious]): *We know that your father has had a very challenging last 2 weeks in the ICU*. *He required immense life* *support* *and as you can see is still recovering*. *You mentioned that before the ICU he was sharp, witty, and could do most daily tasks by himself*. *We certainly hope he makes a speedy recovery home to care for his dogs who he loves taking for daily walks, but we know that patients his age will typically require more assistance after the ICU than before*. *This might mean trouble with performing his favorite crossword puzzles or remembering names of grandchildren*. *These symptoms can have an impact on his quality of life*. *We did our best to minimize the risk of these impairments with the “bundle of care” we discussed each day on rounds*. *With the amount of delirium he suffered, it’s still very likely that he’ll have some degree of problems with his memory, concentration, and decision-making for some time*. *Continuing with his physical and occupational rehabilitation will likely speed his recovery*. *It is worth you being aware and prepared that he will likely need more help around the house and with things like finances or medications for several months and perhaps even years to come*. *Our ICU recovery clinic can provide testing to evaluate any of the residual consequences of his critical illness, and referrals for needed medical or social work services. He is not alone in his recovery*. *Let’s also remember that you as a family have been a part of his suffering, and families experience challenges with mental health struggles just as often as patients*. *Our recovery clinic has a* *support* *group for caregivers like yourself*.

## Conclusion

Communicating PICS to patients and families is paramount in easing the often difficult transition that can occur for individuals who survive the ICU. Patients and families should know about and be part of an A2F bundle approach to improving long-term outcomes. Using empathy and an understanding of risk factors, we advocate for providing awareness of and preparation for PICS while in the ICU. These conversations must continue after hospital discharge with dedicated ICU recovery clinics or primary care.

## Financial/Nonfinancial Disclosures

The authors have reported to *CHEST* the following: M. L. R. is supported by the National Heart, Lung and Blood Institute (Grant No.T32HL087738). E. W. E. is supported by the 10.13039/100000049National Institute on Aging (Grant No. R01AG058969) and the U.S. Department of Veteran Affairs (Grant No. I01RX002992). M. F. M. is supported by the U. S. Department of Veteran Affairs (Grant No. IK2RX004799). C. M. S. is supported by the National Institute of Aging (Grant No. R01AG077644), Patient-Centered Outcomes Research Institute (Grant No. BPS-2022C3-30021), and Department of Defense (Grant No. W81XWH-21-1-0051) None declared (J. C. J., M. E. W).
